# Temporomandibular inflammation mobilizes parvalbumin and FosB/deltaFosB neurons of amygdala and dorsal raphe

**DOI:** 10.1590/1414-431X20209950

**Published:** 2020-06-19

**Authors:** G.C. Nascimento, B.B. de Paula, C.A. Lowry, C.R.A. Leite-Panissi

**Affiliations:** 1 Departamento de Fisiologia, Faculdade de Medicina de Ribeirão Preto, Universidade de São Paulo, Ribeirão Preto, SP, Brasil; 2 Departamento de Psicologia, Faculdade de Filosofia, Ciências e Letras de Ribeirão Preto, Universidade de São Paulo, Ribeirão Preto, SP, Brasil; 3 Department of Integrative Physiology and Center for Neuroscience, University of Colorado Boulder, Boulder, CO, USA

**Keywords:** Amygdala, Dorsal raphe nucleus, FosB, Parvalbumin, Temporomandibular inflammation

## Abstract

Pathophysiological mechanisms involved in orofacial pain and their relationship with emotional disorders have emerged as an important research area for multidisciplinary studies. In particular, temporomandibular disorders (TMD) have been evaluated clinically from both physiological and psychological perspectives. We hypothesized that an altered neuronal activity occurs in the amygdala and the dorsal raphe nucleus (DR), encephalic regions involved in the modulation of painful and emotional information. Adult male Wistar rats were used in an experimental complete Freund's adjuvant (CFA)-induced temporomandibular joint (TMJ) inflammation model. CFA was applied for 1 or 10 days, and the animals were euthanized for brain samples dissection for FosB/ΔFosB and parvalbumin (PV) immunostaining. Our results were consistent in showing that the amygdala and DR were activated in the persistent inflammatory phase (10 days) and that the expression of PV^+^ interneurons in the amygdala was decreased. In contrast, in the DR, the expression of PV^+^ interneurons was increased in persistent states of CFA-induced TMJ inflammation. Moreover, at 10 days of inflammation, there was an increased co-localization of PV^+^ and FosB/ΔFosB^+^ neurons in the basolateral and central nucleus of the amygdala. Different nuclei of the amygdala, as well as portions of the DR, were activated in the persistent phase (10 days) of TMJ inflammation. In conclusion, altered activity of the amygdala and DR was detected during persistent inflammatory nociception in the temporomandibular joint. These regions may be essential for both sensory and affective dimensions of orofacial pain.

## Introduction

One of the most common pain conditions experienced by adults is chronic orofacial pain. Specifically, among the dysfunctions that affect the stomatognathic system, temporomandibular disorder (TMD) associated with myofascial pain is a usual cause for chronic orofacial pain development (1). Preclinical and clinical studies have also elucidated the direct relationship between TMD-induced pain with emotional alterations (2,3).

A consensus has formed regarding the need to clinically evaluate TMDs from both physical and psychological perspectives, and to consider both pharmacological and behavioral treatments (4). However, this idea is not yet widely applied in clinical practice due to the lack of direct preclinical and clinical experimental evidence. Indeed, the affective component of pain is mediated by circuits that are distinct from those mediating the sensory-discriminative component (5) and research on neural mechanisms about emotional behavior in pain models can advance the knowledge on this line of research.

Cellular remodeling of brain structures is directly involved in the pathophysiology of pain-induced emotional disorders, based on findings that cortical and subcortical functional and structural neuroplastic alterations are evident in chronic pain states (6). The amygdala is intensely involved in processing the emotional component of pain, probably through a modulatory role upon major supraspinal pain control areas (7). The nociceptive inputs from extra-amygdaloid areas are processed in the lateral (LA) and basolateral (BLA) nuclei of the amygdala and transmitted to the central nucleus of the amygdala (CEA), which can then modulate pain through connections with the forebrain and brainstem (8). These amygdaloid nuclei are more strongly involved in processing harmful stimuli and are responsible for integrating sensory-affective information in the context of pain (9). Moreover, neurons that express the calcium-binding protein parvalbumin (PV) in the amygdala appear to be subpopulations of GABAergic local circuit neurons (10), constituting 43 and 19% of the populations of PV-positive neurons of GABA-immunoreactive neurons in the BLA and LA, respectively (11).

Also, given the critical role of serotonin in the regulation of affective functions, the serotonergic neurons of the dorsal raphe nucleus (DR) and other raphe nuclei are particularly interesting in the context of the aversion of pain (12). Projections from the DR to many areas are recognized to be involved in pain perception and pain modulation, including the spinal trigeminal nucleus (13) and amygdala (14). Importantly, the GABA interneurons are the most prevalent type of non-5-HT neurons in the DR, occurring mainly in the ventrolateral division of the DR (15).

Regarding both sensory and affective aspects of pain, GABAergic modulation of pain transmission has been extensively investigated in the spinal cord, while its modulatory effect at the supraspinal level is scarce (16). In the context of modulatory effects of GABA signaling at supraspinal levels, the activity of PV-positive GABAergic interneurons within the prelimbic cortex has been shown to alter sensory and emotional pain perception (17).

The inflammatory agents used in pain models range from irritant chemicals (e.g., carrageenan, formalin), microbial cell wall fragments or toxins (e.g., lipopolysaccharide), complete Freund's adjuvant (CFA), and zymosan to agents that directly activate specific receptors on primary sensory neurons (e.g., capsaicin, mustard oil). In particular, CFA has been effectively used for the persistent orofacial pain animal model associated with the temporomandibular joint (TMJ) region (2,18). The physiological factors involved and the duration of the CFA model also make it appropriate for the study of pain in psychiatric comorbidities (19). In this context, our previous results using a CFA animal model showed increased inflammatory indicators (plasma extravasation and neutrophil degranulation), as well as increased nociceptive sensitivity after 1, 3, 7, and 10 days of adjuvant injection into the TMJ of rats (2,20). In the same way, in that same model, an anxiety-like behavior mainly at 1 and 10 days after injections was observed (2).

In the current study, we investigated the role of CFA-induced TMJ inflammation on PV expression in the amygdala and DR, and on neuronal activation of the same regions as assessed by FosB/ΔFosB expression. Fos-B is an immediate-early response gene, an indicator of neuronal activation, and PV is a phenotypic marker for an inhibitory interneuronal population.

## Material and Methods

### Animals

Adult male Wistar rats weighing 250-300 g (total number=28) that were maintained in colony conditions at 24±1°C on a 12-h light/dark cycle (lights on at 06:00 h) with *ad libitum* food and water. All experiments were approved by the Animal Care and Use Committee of the University of São Paulo, at the Ribeirão Preto campus (Brazil) (protocol #11.1.891.53.6). The animals used did not present weight loss throughout the experiments. Moreover, the behavioral data of these animals was previously published (2).

### Complete Freund’s adjuvant injections

Rats were anesthetized with an intramuscular injection of 10% ketamine (75 mg/kg) plus 4% xylazine (10 mg/kg) followed by bilateral intraarticular administration with 50 μg of CFA (*Mycobacterium tuberculosis*, Sigma-Aldrich, USA) suspended in 50 μL paraffin oil or with 50 μL of a 0.9% saline solution (SAL). CFA bilateral injections into the TMJ was adopted based on previous studies (2,21,22 [Bibr B23] [Bibr B24]). The following protocol for the administration of CFA into the TMJ has been described previously by do Nascimento and Leite-Panissi (2).

### Immunochemistry

One or 10 days after administration of CFA bilaterally into the TMJ regions, the animals were deeply anesthetized with 10% ketamine (225 mg/kg) plus 4% xylazine (30 mg/kg) and transcardially perfused with phosphate-buffered saline (PBS) followed by 4% paraformaldehyde in 0.1 M sodium phosphate buffer. The brains were then removed and post-fixed in 4% paraformaldehyde for 2 h. After that, samples were immersed in 30% sucrose in 0.1 M sodium phosphate buffer for cryoprotection for 48 h. Next, brains were cut serially on a cryostat (CM-1900, Leica, Germany) into 30 μm-thick frontal sections and again stored at −70°C until immunochemistry procedure. Free-floating sections were submitted to antigen recovery (heating sections for 30 min in a water bath at 60°C in 0.1 M citrate buffer; pH 6.0). After rinsing in the washing buffer (0.1 M PBS + 0.15% Triton-X100; pH 7.4), the sections were incubated for 30 min at 18°C with 2% hydrogen peroxide (diluted in PBS+Triton) to remove endogenous peroxidase activity. Nonspecific binding sites were blocked by incubation in a solution containing 5% bovine serum albumin plus 5% normal serum (from the species of origin of the secondary antibody) in washing buffer for 1 h at 18°C. Next, these sections were incubated at 18°C with the primary antibody for polyclonal rabbit anti-FosB/ΔFosB antibody (rabbit polyclonal, 1:1000, H-75, sc-7203, Santa Cruz Biotechnology, USA; see 23-25). This antibody recognizes both the transient FosB and the more stable ΔFosB isoforms (25). Alternatively, sections were incubated at 18°C with primary monoclonal mouse anti-PV antibody (mouse monoclonal, 1:1000, P227, Sigma-RBI), as a marker of the PV-positive subset of GABAergic interneurons. After incubation in the primary antibody for 24 h at 18°C, the sections were successively washed (washing buffer) and incubated in biotinylated secondary antibody solution (goat, anti-rabbit/anti-mouse, 1:400, in PBS, Vector Lab, USA) for 90 min at 18°C. Sections were then incubated with the avidin-biotin-peroxidase complex for 2 h at 18°C (Vectastain ABC kit, Vector Lab), and the immunoreactivity was revealed by a peroxidase reaction using diaminobenzidine (Sigma) as the chromogen. Negative control sections were incubated at 18°C in the same way as described above but without primary antibodies.

### Double staining by immunochemistry

A similar protocol (as described above) was used for double immunostaining with the difference of incubation of the primary antibody. The sections were primarily incubated in the first secondary antibody (rabbit anti-FosB/ΔFosB antibody, 1:1000) for 24 h at 18°C, and then washed (washing buffer) and incubated in biotinylated secondary antibody solution (1:400, in PBS) for 90 min at 18°C. Sections were then incubated with the second primary antibody (monoclonal mouse anti-PV antibody, 1:1000) for another 24 h at 18°C and again with biotinylated secondary antibody solution. Finally, sections were incubated with the avidin-biotin-peroxidase complex for 2 h (Vectastain ABC kit, Vector Lab) at 18°C and the immunoreactivity was revealed by a peroxidase reaction using diaminobenzidine (Sigma) as the chromogen.

### Quantification of immunochemistry data

An observer blinded to the treatment conditions quantified the PV^+^ or FosB/ΔFosB^+^ cells immunolabeling. Digital images were obtained in a Leica microscope. Each image was acquired with its bar scale. For quantification, five images were systematically captured for each brain region studied with a 20× objective. Immunoreactive cells were counted using ImageJ software (Research Services Branch, National Institutes of Health, USA). First, a background measure was performed, and this value was removed from the final quantification of all photomicrographs.

Cells with gray (FosB/ΔFosB^+^) or brown (PV^+^) impregnations with areas between 10 and 80 µm^2^ were considered for the counting. Neuroanatomical sites were identified with the help of a stereotaxic atlas of the rat brain by Paxinos and Watson (26).

For double staining quantification, five images from the complete serial section were selected randomly. The number of cells simultaneously stained with FosB/ΔFosB^+^ and PV^+^ in the same areas were counted under microscopy visualization. FosB/ΔFosB and PV double‐labeled neurons were counted, and the proportion of double‐labeling neurons to total FosB/ΔFosB neurons (as percentage) was then calculated.

The anterior-posterior localization from bregma of the analyzed regions were: BLA, CEA, and LA nuclei of the amygdala, -1.72 to -3.24 mm bregma; dorsal division of the dorsal raphe nucleus (DRD), -6.84 to -6.96 mm bregma; ventral division of the dorsal raphe nucleus (DRV), -7.20 to -8.40 mm bregma; and lateral division of the dorsal raphe nucleus (DRL), -7.20 to -8.04 mm bregma.

### Statistical analysis

Statistical differences between means were calculated using a two-way ANOVA for repeated measures followed by the *post hoc* Newman-Keuls test, with treatment and time as factors for statistical analysis. Data were considered statistically significant when P<0.05.

## Results

**Figure 1:**
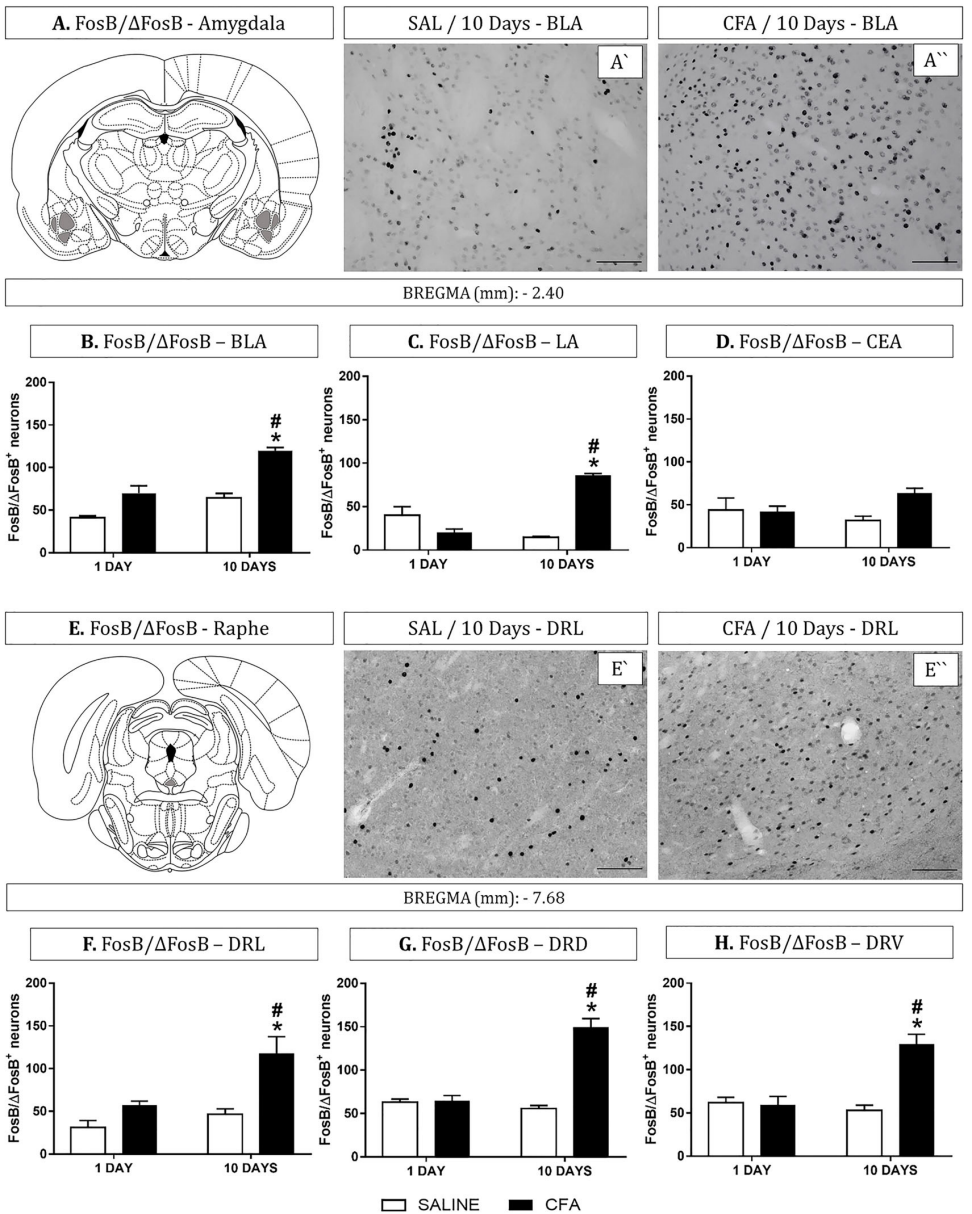
Subregions of the amygdala and dorsal raphe nucleus were analyzed for neuronal activation in complete Freund's adjuvant (CFA)-induced temporomandibular inflammation (1 or 10 days) and rats administered saline (SAL) (n=6-7 per group). **A**, Plate adapted from Paxinos and Watson Atlas (26; Elsevier Academic Press) of basolateral nucleus of the amygdala (BLA); **A'**, photomicrograph of FosB/ΔFosB staining in the BLA of a representative rat from the Day 10/saline-treated group and **A''**, from the Day 10/CFA-treated group. Graphs show FosB/ΔFosB immunohistochemistry (IR) quantification in BLA - **B**, lateral (LA - **C**), and central nuclei of the amygdala (CEA - **D**) (-2.40 mm bregma). **E**, Plate adapted from Paxinos and Watson Atlas (26; Elsevier Academic Press) of the ventral division of the dorsal raphe (DRV); **E'**, photomicrograph of FosB/ΔFosB IR in the DRL of a representative rat from the Day 10/saline treated group and **E''**, from the Day 10/CFA treated group. Graphs show FosB/ΔFosB IR quantification in lateral (DRL - **F**), dorsomedial (DRD - **G**), and ventral divisions (DRV - **H**) of the dorsal raphe nucleus (-7.68 bregma). Data are reported as means±SE. *P<0.05 *vs* Day10/control group (saline), ^#^P<0.05 *vs* Day 1/CFA group (Newman-Keuls test). Magnification, 20×; scale bars, 100 µm.

The effects of CFA-induced TMJ inflammation on FosB/ΔFosB expression in the distinct nuclei of the amygdala and DR are presented in Figure 1. In the BLA, CFA injections led to a significant increase of FosB/ΔFosB positive neurons. The group with 10 days of CFA-induced inflammation was different compared with Day 10/saline and Day 1/CFA-induced inflammation groups (P<0.05, Figure 1B). The LA analysis also revealed an increase in FosB/ΔFosB^+^ neurons in the group with 10 days of CFA-induced inflammation and was different compared with Day 10/saline and Day 1 of CFA-induced inflammation groups (P<0.05, Figure 1C). However, no difference was found in FosB/ΔFosB^+^ neurons in the CEA among groups (Figure 1D).

In the DRL, we found significant effects on the number of FosB/ΔFosB^+^ neurons in the group with 10 days of CFA-induced inflammation compared with Day 10/saline and Day 1/CFA-induced inflammation groups (P<0.05, Figure 1F). Similarly, in the DRD, an increase of FosB/ΔFosB^+^ neurons in the group with 10 days of CFA-induced inflammation was found compared with Day 10/saline and Day 1 of CFA-induced inflammation groups (P<0.05, Figure 1G). FosB/ΔFosB^+^ neurons in the DRV showed a significant increase in the group with 10 days of CFA-induced inflammation compared with Day 10/saline and Day 1 of CFA-induced inflammation groups (P<0.05, Figure 1H).

**Figure 2:**
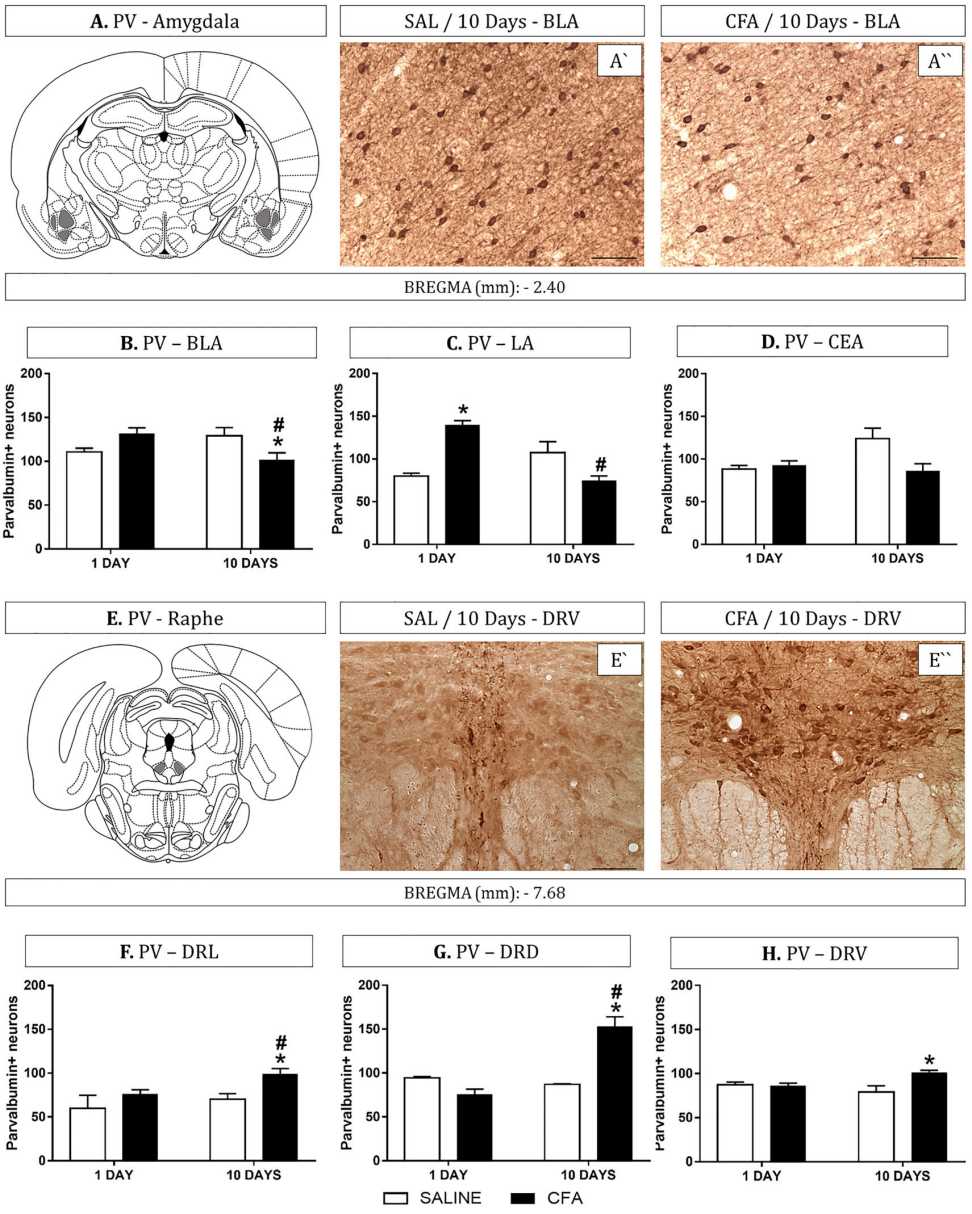
Subregions of the amygdala and dorsal raphe nucleus were analyzed for parvalbumin (PV) expression in complete Freund's adjuvant (CFA)-induced temporomandibular inflammation (1 or 10 days) and rats administered saline (SAL) (n=6-7 per group). **A**, Plate adapted from Paxinos and Watson Atlas (26; Elsevier Academic Press) of basolateral nuclei of the amygdala (BLA); **A'**, photomicrograph of PV staining in the BLA of a representative rat from the Day 10/saline-treated group and **A''**, from the Day 10/CFA treated group. Graphs show PV immunohistochemistry (IR) quantification in basolateral (BLA - **B**), lateral (LA - **C**), and central nuclei of the amygdala (CEA - **D**) (-2.40 mm bregma). **E**, Plate adapted from Paxinos and Watson Atlas (26; Elsevier Academic Press) of the lateral division of the dorsal raphe nucleus (DRL); **E'**, photomicrograph of PV IR in the DRV of a representative rat from the Day 10/saline-treated group and **E''**, from the Day 10/CFA-treated group. Graphs show PV IR quantification in lateral (DRL - **F**), dorsal (DRD - **G**), and ventral (DV - **H**) divisions of the dorsal raphe nucleus (-7.68 mm bregma). Data are reported as means±SE. *P<0.05 *vs* Day 10/control group (saline), ^#^P<0.05 *vs* Day 1/CFA group (Newman-Keuls test). Magnification, 20×; scale bars, 100 µm.

The amygdala and DR analyses revealed that CFA-induced TMJ inflammation produced an alteration of PV^+^ interneuron expression (Figure 2). Among PV^+^ neuron counts in the BLA, we found a significant reduction of the group with 10 days of CFA-induced inflammation compared with Day 10/saline and Day 1 of CFA-induced inflammation groups (P<0.05, Figure 2B). Interestingly, in the LA analysis, the group with 1 day of CFA-induced inflammation showed more PV^+^ neurons compared with the Day 1/saline group. However, 10 days of CFA-induced inflammation lead to a decrease in the PV^+^ neurons compared with Day 1 of CFA-induced inflammation group (P<0.05, Figure 2C). In the CEA, there was no significant effect on PV^+^ neurons among the groups (P<0.05, Figure 2D).

In the DRL, CFA significantly increased the number of PV^+^ neurons in the group with 10 days of CFA-induced inflammation and was different compared with the Day 10/saline group (P<0.05, Figure 2F). In the DRD, 10 days of CFA-induced inflammation significantly increased the PV^+^ neurons compared with Day 10/saline and Day 1 of CFA-induced inflammation groups (P<0.05, Figure 2G). Regarding DRV, CFA increased the PV^+^ neuron counts in the group with 10 days of CFA-induced inflammation compared with the Day 10/saline group (P<0.05, Figure 2H).

**Figure 3:**
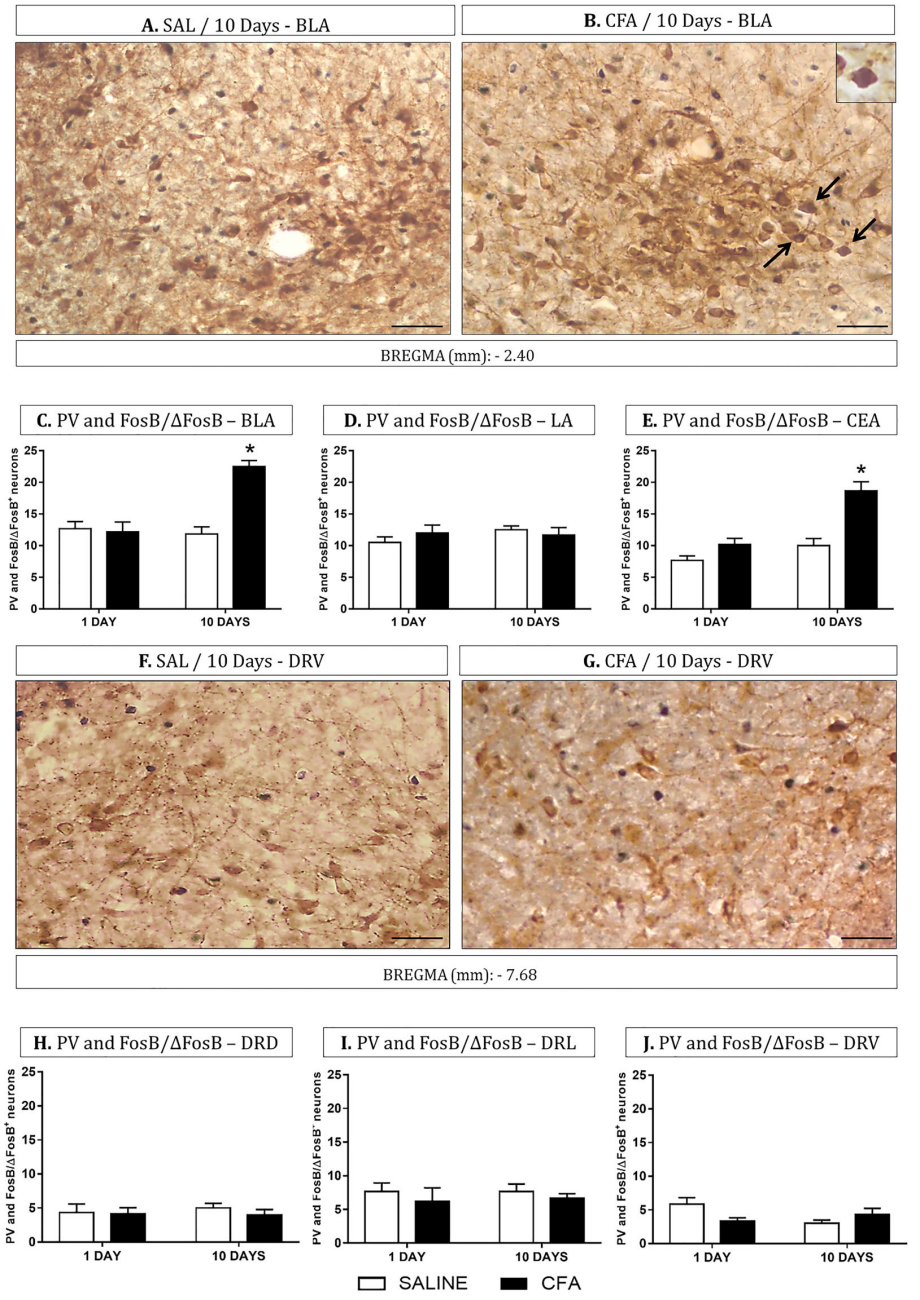
Representative photomicrographs of co-localization of FosB/ΔFosB over the entire neuronal population of parvalbumin (PV), or unlabeled cells in the amygdala (**A**-**E**) and dorsal raphe (**F**-**J**) (n=6 per group). SAL: saline; CFA: complete Freund's adjuvant. (**C**-**E**) Percent of co-localized PV and FosB/ΔFosB^+^ neurons in basolateral (BLA), lateral (LA), and central nuclei of the amygdala (CEA). (**H**-**J**) Percent of co-localized PV and FosB/ΔFosB^+^ neurons in the dorsal (DRD), lateral (DRL), and ventral (DRV) divisions of the dorsal raphe nucleus. Black arrows indicate FosB/ΔFosB^+^ (gray nucleus), PV^+^ (brown cell body), and unlabeled (cell body not labeled) neurons. Data are reported as means±SE. *P<0.05 *vs* Day 1/CFA group and Day 10/control group (saline) (Newman-Keuls test). Magnification, 40×; scale bars, 100 µm.

Double staining of FosB/ΔFosB and PV neurons were made in the amygdala and DR (Figure 3). In the BLA, the group with 10 days of CFA-induced inflammation showed an increase in the FosB/ΔFosB and PV^+^ neurons compared with Day 10/saline and Day 1/CFA-induced inflammation groups (P<0.05, Figure 3C). Moreover, the CEA analysis also revealed an increase in the FosB/ΔFosB^+^ and PV^+^ neurons in the group with 10 days of CFA-induced inflammation compared with Day 10/saline and Day 1 of CFA-induced inflammation groups (P<0.05, Figure 3E). Additionally, no effect was found in the co-localization of PV^+^ and FosB/ΔFosB^+^ neurons on LA and DR (Figure 3D and H-J).

## Discussion

Our findings showed that: i) different nuclei of the amygdala, as well as portions of the DR, were activated in the persistent phase (10 days) of TMJ inflammation; ii) the expression of PV^+^ interneurons in the amygdala was decreased while in the DR it was increased in persistent states of CFA-induced TMJ inflammation; and iii) in the acute phase of CFA-induced TMJ inflammation (i.e., Day 1), these changes were not detected.

Indeed, chronic pain is a complex web of sensory and emotional experiences, coupled with behavioral adaptations (27) and, in particular, chronic orofacial pain involves risk factors such as stress and depression, as well as physical dysfunction (28). Psychological stress that perpetuates and worsens is a risk factor for temporomandibular disorders of muscular or articular origin (29).

We demonstrated here that the two structures frequently recruited in the emotional and affective dimensions of nociception suffer alterations in the persistent phase of CFA-induced TMJ inflammation, an established experimental model for temporomandibular inflammatory nociception as reported previously (2). Importantly, changes in the amygdala neural circuit have been detected in different preclinical models of pain and linked mechanistically to pain behaviors, suggesting that these maladaptive neuroplastic changes are a brain mechanism of pain (7).

CFA-induced chronic pain is a potent experimental model for investigations about specific neural circuits remodeling involved in chronic pain and its comorbidity (30). Recent findings (30) suggest that the nociceptive stimuli evoked by CFA intraplantar inflammation for 7 days could weaken the excitatory glutamatergic information to the indirect pathway within the reward circuits (nucleus accumbens), indicating that the mesolimbic reward circuitry is critical for pain sensation and pain-related emotional experiences. Again, the CFA-injected mouse model is used to induce pain and anxiety-like behaviors synchronously. In accordance, Guan et al. (31) administered CFA in the hind paw to produce pain- and anxiety-like behaviors, and 7 days later, they observed the anxiolytic effects of polydatin by suppressing inflammatory cytokines in the amygdala in this model. Corroborating such findings, our previous data are consistent in suggesting the CFA-experimental model for temporomandibular inflammatory nociception for 10 days as generating an anxiolytic-like response (2).

Highly processed information generated in the LA-BLA network is transmitted to the CEA, which serves major amygdala output functions and is a locus of the descending inhibitory pathway, through forebrain and brainstem connections (32). Of note, it has been shown that the activated neurons in the CEA allow the descending inhibitory pain pathway to be disinhibited (33). Accordingly, our data showed an increased FosB/ΔFosB expression in the CEA at 10 days of TMJ inflammation. Importantly, excitatory neurons in the cortex are under strong regulation by GABAergic interneurons, and previous findings have shown that optogenetic stimulation of one class of GABAergic interneurons, i.e., PV-containing interneurons, is sufficient to have a substantial effect on pain thresholds (34). In the study by Kang and colleagues (34), the stimulation of this single interneuron class mimicked the inhibition effects of the excitatory neurons within this brain structure during painful stimuli, and PV activation was able to alleviate mechanical hypersensitivity. Our results corroborate these findings implicating the PV^+^ subset of GABAergic interneurons in control of chronic pain since we found a decreased expression of PV^+^ neurons after 10 days of TMJ inflammation compared to control and 1 day of inflammation in the BLA, and after 1 day of inflammation in the LA. However, we did not find a reduction of the PV^+^ neurons in the CEA after 10 days of TMJ inflammation; the percentage of co-localized PV^+^ and FosB/ΔFosB^+^ neurons were higher in the 10 days of TMJ inflammation compared to controls and after 1 day of inflammation in CEA and BLA. It has been shown that one week following peripheral nerve injury was enough to decrease PV interneuron activity in the somatosensory cortex (35), and the optogenetic activation of these cells promoted a reduction of the CFA-induced mechanical hypersensitivity (34). Our results are consistent with these studies since we found that 10 days of CFA-induced inflammation led to a reduction of PV^+^ neurons in the BLA and LA. However, the increase of the co-localization of the PV^+^ and FosB/ΔFosB^+^ neurons in the BLA and CEA shows that these interneurons are being recruited in the chronic phase of TMJ inflammation.

The brainstem DR is involved in the descending pathway that controls noxious inputs to the spinal cord, as well as noxious inputs to the trigeminal complex (36), and it participates in the normal physiological responses to noxious stimulation (37). This inhibition of the nociceptive responses might be partly achieved by direct modulation of spinal/trigeminal nociceptive transmission by the DR (38). The activation of this structure during orofacial inflammation, as shown by our results, demonstrated the adaptation of this complex system in response to chronic orofacial inflammation and may suggest an ineffective nociception modulation. In respect to the role of the DR in pain modulation, functionally, GABAergic signaling mechanisms have been described to be hyperalgesic in this region, since microinjection of a GABA receptor agonist into the DR antagonizes the analgesia produced by morphine microinjected at the same site (38). Our data were consistent in showing increased expression of PV^+^ neurons in the DR in the persistent phase of CFA-induced TMJ inflammation, indicating that these inhibitory interneurons in these structures may be involved in orofacial inflammation modulation.

An important point to be considered is the neuroplasticity of brain function and structure that individuals who experience chronic orofacial pain can present. Brain neuroimaging has helped the investigation on this topic (39,40). The prefrontal cortex, the limbic system, and the circuitry of reward processing appear to be functionally and structurally altered during chronic orofacial pain (40). The neural mechanisms of such adaptive alterations in brain organization regarding temporomandibular chronic inflammation are still unclear. Our data add to the information on molecular changes in the amygdala and raphe that may be indicative of such plasticity and can contribute to brain markers of chronic orofacial pain.

For the first time, we showed the neuronal activation of the different nuclei of the amygdala and DR on TMJ inflammation. This finding can help clarify the different neural networks and will be essential for the future development of novel therapies for treating physical and emotional aspects of orofacial pain disorders in patients.
